# Oxidative stress in the tumor microenvironment in gastric cancer and its potential role in immunotherapy

**DOI:** 10.1002/2211-5463.13630

**Published:** 2023-05-20

**Authors:** Yu Yu, Yingjie Wu, Yixing Zhang, Mengxiao Lu, Xiaobao Su

**Affiliations:** ^1^ Department of Gastrointestinal Minimally Invasive Surgery The Affiliated People's Hospital of Ningbo University China

**Keywords:** gastric cancer, immunotherapy, oxidative stress, T cells, tumor microenvironment

## Abstract

Gastric cancer (GC) is the fourth leading cause of cancer‐related death and the fifth most common malignant tumor globally. However, the clinical efficacy of conventional therapies is limited. Currently, immunotherapy is considered an effective therapeutic strategy for the management of various cancers, especially GC, but is of only limited benefit for GC patients. Accumulating evidence has revealed that oxidative stress plays a critical role in the regulation of immune responses within the tumor microenvironment (TME), affecting the efficacy of immunotherapies. Reactive oxygen species exert critical roles in enhancing antigen presentation, regulating immune responses, and preventing immunoescape. In this review, we summarize the dominant cancer immunotherapeutic strategies and describe the interaction between oxidative stress and the immune TME. We emphasize the underlying mechanisms of the efficacy of cancer immunotherapy, which involves its effects on oxidative stress, in the context of GC. We also highlight the therapeutic potential of regulating oxidative stress to improve immunotherapies, which may have benefits for clinical practice.

## Graphical abstract

Predominant reactive oxygen species‐modulating agents are used for cancer immunotherapy, and the potential mechanisms of certain pro‐oxidants enhance the efficacy of cancer immunotherapies.

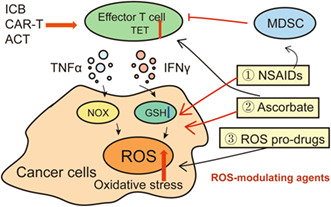

**Abbreviations**
8‐OHdG: 8‐hydroxydeoxyguanosine
ACT: adoptive cell therapy
APCs: antigen‐presenting cells
CAR: chimeric antigen receptor
CAR‐Ts: chimeric antigen receptor T cells
COXs: cyclooxygenases
CRC: colorectal cancer
CSCs: cancer stem cells
CVs: cancer vaccines
DAMPs: damage‐associated molecular patterns
DCs: dendritic cells
ECM: extracellular matrix
EMT: epithelial‐to‐mesenchymal transition
ER: endoplasmic reticulum
ETC: electron transport chain
GC: gastric cancer
HCV: hepatitis C virus
HIF1a: hypoxia‐inducible factor‐1
ICB: immune checkpoint blockade
ICD: immunogenic cell death
ICIs: immune checkpoint inhibitors
NAC: *N*‐acetyl cysteine
NFAT: nuclear factor of activated T cells
NK cells: natural killer cells
NOXs: NADPH oxidases
NRF2: erythroid 2–related factor 2
NSAIDs: nonsteroid anti‐inflammatory drugs
OS: overall survival
OVs: oncolytic viruses
PGE2: prostaglandin E2
PTEN: phosphatase and tensin homolog
RNS: reactive nitrogen species
TAMs: tumor‐associated macrophages
TCR‐Ts: T‐cell receptor T cells
TET: ten‐eleven translocation
TME: tumor microenvironment
Tregs: regulatory T cells

Gastric cancer (GC) is the fourth leading cause of cancer‐related death and the fifth most common malignant tumor globally [1]. Currently, the standard treatment for advanced GC is combination regimens involving a fluoropyrimidine or a platinum agent with paclitaxel [2]. However, with advanced treatment, the median survival of GC remains 12–15 months. Therefore, there is an urgent need to establish novel effective therapeutic strategies for treating GC [3].

During the past decades, accumulating studies have investigated the possibility of applying immune responses to kill cancer cells in patients, which is known as cancer immunotherapy [4, 5, 6, 7, 8]. However, due to the immunosuppressive mechanisms of tumor cells, few patients benefit from cancer immunotherapies [9, 10]. Tumor cells can release a variety of suppressive cytokines or immunosuppressive molecules once their antigens are recognized by immune cells, leading to the weak antitumor function of immune cells, which is called immune escape. Moreover, tumor cells can recruit suppressive cells or suppressive cytokines [11]. According to previous studies, these responses of cancer cells are closely associated with reactive oxygen species (ROS)‐induced oxidative stress [12]. ROS are a group of highly reactive oxygen‐containing molecules that have pathologically elevated levels in various cancers or patients exposed to chemoradiotherapy [13, 14, 15]. Given the multifaceted role of ROS in biological function, the balance between ROS production and scavenging should be highly controlled, since the disruption of this balance leads to oxidative stress.

In this review, we describe the role of oxidative stress within the tumor microenvironment (TME). Moreover, we summarize the existing cancer immunotherapy strategies and discuss the interaction between oxidative stress and immunotherapies. Based on these findings, we emphasize the therapeutic potential of a rational combination of methods that manipulate oxidative stress to enhance cancer immunotherapies, which may be a novel and useful approach in the clinical treatment of GC with great efficacy and limited side effects.

## Origin of oxidative stress

It has been reported that *Helicobacter pylori* produces superoxide anions (O_2_^−^) to suppress the bactericidal function of immune cells. In addition, the production of O_2_^−^ can be significantly inhibited by cyanide (CN^−^), suggesting that the production of O_2_^−^ might be passively caused by electrons leaking from the mitochondrial respiratory chain of *H. pylori* [16, 17]. The cytotoxicity of O_2_ is moderate, while the cytotoxicity of hydroxyl radicals (^·^OH), which are produced through Fenton's reactions, is much higher. One of the underlying mechanisms of *H. pylori*‐induced carcinogenesis involves the accumulation of oxidative DNA damage. 8‐Hydroxydeoxyguanosine (8‐OHdG) is the predominant oxidatively modified product of DNA. According to previous studies, higher levels of 8‐OHdG can be observed in tumor tissues or adjacent areas than in normal tissues in patients with GC [18, 19]. Excessive oxidative DNA damage precedes DNA repair, resulting in DNA mutation. Moreover, intracellular accumulation of ROS/reactive nitrogen species (RNS) can trigger DNA mutation by disrupting the expression of various tumor‐suppressing genes and ultimately contribute to gastric carcinogenesis [20, 21].

Reactive oxygen species are the natural byproducts of electron transport chain activity (ETC) [22]. Superoxide production is caused by incomplete electron transfer and leakage through ETC complexes. Another source of ROS is membrane‐bound NADPH oxidases (NOXs) [23]. The production of superoxide from O_2_ and NADPH can be accomplished by NOXs. In addition, there are seven NOX isoforms in most mammals [24]. Previous studies have illustrated that the predominant gastroduodenal alterations in *H. pylori*‐infected patients are caused by ROS and RNS. Excessive ROS/RNS production can be observed in *H. pylori*‐infected gastric mucosa, which is also associated with mucosal damage and further carcinogenesis. Moreover, cytotoxins derived from *H. pylori*, including CagA, trigger mitochondrial production of ROS in GC [25].

## The effects of oxidative stress on GC cells

Accumulating evidence has revealed that higher levels of oxidative stress, which is associated with an elevated presence of ROS, can be observed in GC cells than in normal tissues [26]. It has been demonstrated that mitochondria are the predominant organelles responsible for the production and release of ROS. Of note, a variety of intracellular events, including oncogenesis, inactivation of tumor suppressor genes, abnormal metabolism, and resistance to hypoxia, can cause deregulated ROS generation in cancer cells, especially in GC [27].

### The role of oxidative stress in GC carcinogenesis

Accumulating evidence has demonstrated that high levels of ROS can contribute to tumor progression by promoting cell proliferation, migration, survival, and therapeutic resistance. A variety of growth factor signaling pathways and gene mutations are responsible for the elevated production of ROS, which is one of the predominant factors involved in carcinogenesis, especially in GC [28]. According to previous studies, a wide range of growth factors are involved in this progression. To date, it has been reported that there are three critical signaling pathways mediating oncogene‐induced ROS production. The first is PI3K/Akt/mTOR signaling, which plays a crucial role in cell survival [29]. The increase in ROS levels can activate this signaling by inactivating its negative phosphatase regulators, such as phosphatase and tensin homolog (PTEN) [30]. It has been demonstrated that the inactivation of PTEN can be frequently observed in GC, indicating that ROS‐induced activation of PI3K/Akt/mTOR signaling plays an important role in GC progression [31]. The other mediator is the MAPK signaling pathway, which is responsible for cell proliferation and survival. Similarly, ROS‐induced MAPK signal activation is mediated by oxidizing and inactivating MAPK phosphatases [32]. Finally, ROS can also degrade the phosphatase inhibitor of NF‐κB and then induce NF‐κB translocation. As a critical transcription factor responsible for inflammation, cell proliferation, and survival, NF‐κB plays an important role in carcinogenesis, tumor growth, and metastasis in GC [33, 34].

### The role of oxidative stress in the angiogenesis and metastasis of GC

Accumulating evidence has also revealed the role of elevated ROS levels in angiogenesis, metastasis, and therapeutic resistance. It has been demonstrated that angiogenesis is partially caused by ROS [32]. During the progression of tumor growth, tumor cells are deprived of oxygen due to inadequate blood supply, which is called hypoxia [35]. To meet the increased metabolic needs of cells, the production of new blood vessels, known as angiogenesis, is triggered. Hypoxia induces the production of mitochondrial ROS, which stabilizes hypoxia‐inducible factor‐1 (HIF1a) and then promotes cell survival and tumor progression [36]. Moreover, it has been revealed that cancer metastasis is another feature of gastric malignancy. Epithelial‐to‐mesenchymal transition (EMT) is the predominant cause of tumor metastasis and involves the transition of epithelial cells into mesenchymal cells [37]. Based on a previous study, ROS induce the expression of metalloproteinases and mediate the degradation of the extracellular matrix (ECM), leading to the further occurrence of EMT [38].

By contrast, the excessive production of ROS can also induce the cell death of cancer cells [39]. However, cancer cells also present an elevated antioxidant capacity to resist oxidative stress conditions [40]. A wide range of endogenous antioxidant enzymes, including superoxide dismutase, glutathione reductase, and catalase, mediate the function of GC. In addition, cancer stem cells (CSCs) have a less differentiated but highly tumorigenic role in the progression of GC [41, 42]. CSCs exhibit heightened antioxidant capacity, which is responsible for their resistance against ROS‐mediated oxidative damage and chemoradiotherapies [43]. Moreover, transcription factors, especially nuclear factor erythroid 2–related factor 2 (NRF2), play a critical role in the regulation of redox homeostasis. Excessive production of ROS inhibits the degradation of NRF2, resulting in the initiation of the transcription of various antioxidant genes. Therefore, NRF2 is considered an alleviator of excessive oxidative stress and promotes tumor growth and progression under conditions of imbalanced redox status in the TME in GC [44]. Due to the critical role of NRF2 in tumor growth and therapeutic resistance, NRF2 may be a potentially effective target for anticancer therapeutic strategies in future.

## The role of oxidative stress in the immune TME within GC

The TME is a dynamic environment in GC that consists of a variety of cells, including cancer cells, immune cells, fibroblasts, blood vessels, stromal cells, and ECM [45]. Tumor cells interact with various cells in the TME and further establish a protumoral microenvironment [46]. ROS can mediate cell death by infiltrating macrophages and neutrophils. Additionally, ROS mediate the activation of T lymphocytes and natural killer (NK) cells, which are responsible for immunosurveillance and cytotoxicity [47]. By contrast, increased ROS concentration is also associated with the function of various tumor‐promoting immune cells, such as regulatory T cells (Tregs) and tumor‐associated macrophages (TAMs), which are intimately connected with the progression of GC [26, 48]. By influencing the dynamic TME, GC cells establish an immunosuppressive microenvironment and present immune tolerance through ROS. In the following section, we describe the predominant role of ROS in mediating the effects of immunotherapies on various immune cells in the treatment of GC.

### Oxidative stress and immunosurveillance: T lymphocytes and NK cells

The imbalance of oxidation/antioxidation in GC patients leads to mitochondrial dysfunction, apoptosis, and abnormal immune function of T lymphocytes and NK cells [49]. Many studies have reported that the activation of T lymphocytes is partially induced by elevated ROS levels. ROS are considered the second messengers that initiate the nuclear factor of activated T cells (NFAT) and downstream signaling pathways [50, 51]. In turn, the activation of both T lymphocytes and NK cells also leads to further production of ROS. ROS are also responsible for the regulation of immune homeostasis through the induction of cell death. According to a previous study, ROS promoted the increased expression of FAS and a decrease in BCL2 levels to induce the apoptosis of T lymphocytes [52]. Therefore, maintaining the delicate control of ROS levels in T lymphocytes and NK cells is extremely important. It has been demonstrated that excessive ROS levels in T and NK cells can decrease TCR and CD16 chain levels and block NF‐kB activation, resulting in deficient IFNγ, TNFα, and IL‐2 production and impaired metabolic integration and reprogramming during inflammatory T‐cell responses [53, 54]. Among various regulatory mechanisms, the GSH pathway plays a critical antioxidative role in the redox status in T lymphocytes [55]. GSH deficiency in T lymphocytes leads to the decreased expression of NFAT and eventually impaired metabolic integration and biological function [56]. In an experimental study, after stimulation with *N*‐acetyl cysteine (NAC), reduced DNA damage and apoptosis were observed in T lymphocytes [57]. Moreover, GC‐bearing mice presented increased antitumoral features after the injection of NAC‐treated T lymphocytes [58]. The expression of chimeric antigen receptor (CAR) and catalase in T lymphocytes counteracts the imbalanced redox status in T lymphocytes, prevents ROS‐mediated inhibition of NK cells, and further presents antitumoral effects in GC [59, 60]. Similarly, NK cells acquire resistance against the effects of disturbed redox status by IL‐15 and protect other immune cells from ROS‐mediated apoptosis within the TME of GC [61].

### Oxidative stress and antigen presentation: APCs

Appropriate ROS levels are also required for the maintenance of the biological function of antigen‐presenting cells (APCs) within the TME of GC [62]. It has been demonstrated that antigen presentation in macrophages and dendritic cells (DCs) is mediated by NOX2‐induced phagosomal ROS [63]. Extracellular ROS can also modify the immunogenicity of antigenic peptides, altering T‐cell priming [64]. In addition, the initiation of oxidative stress in the endoplasmic reticulum (ER) leads to immunogenic cell death (ICD) in various cancers, especially in GC [65]. The ICD of cancer cells results in the release of a variety of harmful substances, including damage‐associated molecular patterns (DAMPs) [66]. The interaction between DAMPs and their responding receptors (CD91 for CRT, TLR4 for HMGB1, P2RX7 for ATP) significantly enhances the activation of DCs and antigen presentation. According to previous studies, the administration of antioxidants attenuated ICD, while increased levels of ROS in the ER induced ICD and promoted antitumoral immunity. Of note, it has also been highlighted that the production and duration of elevated ROS levels determine the occurrence of ICD and effective antitumor immunity [67].

### Oxidative stress and immunosuppressive cells in the TME

In addition to the ROS‐mediated antitumoral function, the production of ROS induces the activation of Tregs and further immunosuppressive function in the TME of GC [68]. Increased infiltration by Tregs can be observed in GC, suggesting the persistence of Tregs with excessive ROS within the TME [69]. A previous study revealed that GSH deficiency in Tregs promoted cell proliferation and immunosuppressive function, indicating its great resistance to imbalances in redox status [70]. Moreover, after Tregs undergo apoptosis, ATP is transformed into adenosine, which significantly suppresses immune checkpoint blockade (ICB)‐induced antitumor immunity [71]. Of note, a variety of myeloid cells, including neutrophils, macrophages, and MDSCs, are reported to produce excessive ROS within the TME. In particular, phagocytic cells release ROS and present antitumor effects after chemoimmunotherapy [72]. In addition, neutrophils and MDSCs produce ROS or RNS and mediate the immunosuppressive function of T lymphocytes. Of note, high ROS levels within the TME promote the immunosuppressive status of MDSCs, while NOX2 deficiency or inhibition of oxidative stress triggers the differentiation of myeloid cells into TAMs [73].

According to a previous study, TAMs are the most abundant immune cells within the TME and are closely associated with the progression of various cancers. Based on their specific biological function, TAMs can be classified into the antitumoral subtype (M1) and protumoral subtype (M2). Therefore, reprogramming TAMs to the antitumor phenotype may be an effective strategy to enhance the efficacy of immunotherapy [74]. Moreover, it has been reported that increasing ROS levels within the TME can promote the shift of M1‐like TAMs into M2 subtypes [75]. In cocultures with neuroblasts, increased lipid biosynthesis can be observed in TAMs. In one study, tumor‐stimulated macrophages were more capable of producing ROS than control macrophages [76]. However, in melanoma, after treatment with high ROS levels, TAMs presented a more invasive phenotype, involving the secretion of TNFα [77]. Notably, high PD‐L1 expression also promotes the M2‐like polarization of TAMs [78]. Additionally, TAMs acquire an immunosuppressive phenotype and increase PD‐L1 expression when treated with inducers of ROS production. Mechanistically, the accumulation of ROS activates NF‐κB signaling to promote the transcription of PD‐L1 and the release of immunosuppressive chemokines. Therefore, ROS may regulate PD‐L1 expression by altering the macrophage M1/M2 balance [79].

## The new era of clinical immunotherapy for GC

**Fig. 1:**
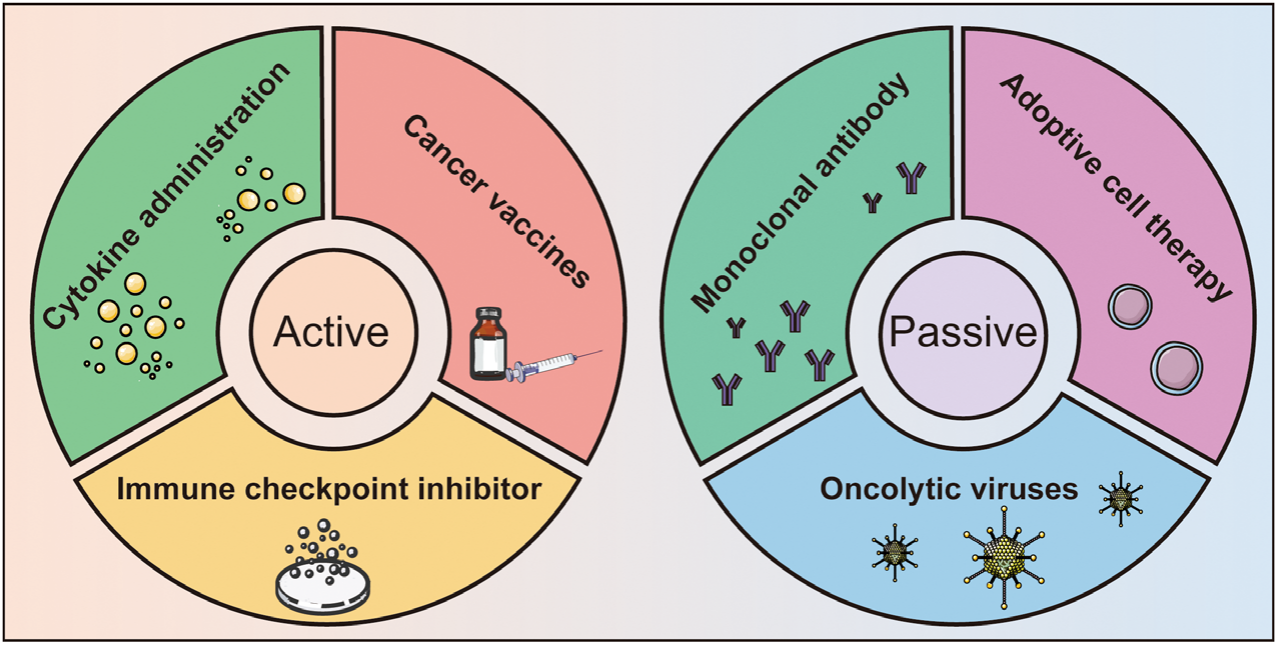
Classification of existing cancer immunotherapy. Immunotherapy is categorized as active or passive subtypes.

Due to the underlying mechanisms of tumor immune escape, cancer immunotherapies have been well established in recent decades with great clinical value. Cancer immunotherapy can be divided into two dominant subtypes: active or passive immunotherapies [8]. Active immunotherapies are based on the enhanced antitumor immune response in the host and include immune checkpoint inhibitors (ICIs), cancer vaccines (CVs), and cytokine administration. Alternatively, passive immunotherapies can directly present tumor‐killing function through the injection of effector cells or antibodies [80]. According to previous clinical studies, after the administration of immunotherapies, durable and curative outcomes can be observed in many patients with GC. In the following section, we summarize the existing advanced immunotherapeutic strategies for GC (Fig. 1).

### Active immunotherapies

Immune checkpoint inhibitors are one of the most predominant cancer immunotherapies in clinical practice. Immune checkpoints are responsible for maintaining appropriate immune responses and preventing the immune attack of normal tissues. However, tumor cells take advantage of immune checkpoints to escape from the immune response. Therefore, ICIs were established to block the binding of checkpoints and recover antitumor immunity within the TME [81]. To date, PD‐1/PD‐L1 blockade and CTLA4 inhibitors are the predominant ICIs used in clinical practice [82, 83, 84]. ICB treatment is based on the inhibition of PD‐1 and CTLA4 and facilitates the activation of T lymphocytes and biological function. Tumor cells express PD‐L1 to bind to the PD‐1 receptors in T cells to avoid T‐cell‐based elimination, whereas the administration of PD‐1/PD‐L1 blockade disrupts the binding and induces cell death in cancers [85]. In the phase III ATTRACTION‐2 trial, an anti‐PD‐1 monoclonal antibody, nivolumab, significantly improved overall survival (OS) in patients with GC after chemotherapy, which led to the approval of nivolumab for the treatment of GC in Asian countries [86]. CTLA4 is another immune checkpoint that regulates the activation of T lymphocytes. The interaction of CD80/86 and CTLA4 inhibits T‐cell activation and promotes tumor progression in various cancers, especially in GC [87]. Therefore, targeting CTLA4 is another promising approach for the treatment of cancers [88]. Cytokine treatment is the first active immunotherapy that was applied in clinical practice. Cytokines are small molecules released to regulate immune responses. Therefore, direct injection of cytokines can boost the immune responses within the TME. Interferons can induce the maturation of immune cells, such as NK cells and macrophages, and further trigger immune responses during the treatment of GC. In addition, IL‐12 has been applied for the treatment of various types of cancers, especially GC [89]. CVs are another effective therapeutic strategy to initiate immune responses to kill cancer cells [90]. Prophylactic vaccines can reduce the incidence of various cancers. Currently, several vaccines can efficiently inhibit tumor progression in clinical practice.

### Passive immunotherapies

Monoclonal antibodies were the first validated immunotherapeutic strategy. Antibody administration can activate apoptosis and antibody‐dependent cellular cytotoxicity by immune cells [91]. Conjugated antibodies are another effective approach; these antibodies carry active factors and deliver effector molecules directly to cancer cells. Oncolytic viruses (OVs) exist in nature or are genetically engineered to selectively replicate in cancer cells to enhance immune responses and trigger tumor cell lysis [92]. The selective replication of OVs in cancer cells directly induces tumor lysis. Moreover, the promoted release of tumor‐associated antigens or cytokines from cancer cells aids in the elimination of cancers by OVs [93]. Recently, a variety of OVs, including Herpeviridae, reovirus, adenoviridae, and several RNA viruses, have been applied in clinical trials. Reovirus is an OV isolated from the respiratory tract or intestinal tract of humans that have a natural preference for cancer cells [94]. It has been demonstrated that serotype 3 reovirus presented oncolytic activity in multiple tumor types. Moreover, reovirus can stimulate the immune system to kill liver cancer cells and clear hepatitis C virus (HCV) [95]. OVs also have a synergistic effect on killing tumor cells with currently used single‐target anticancer drugs. It has been proven that clinical‐grade oncolytic orthoreovirus (Reo) elicits innate immune activation within primary human liver tissue in the absence of cytotoxicity and independently of viral replication. In addition to achieving therapy for HCC through innate degranulating immune cells, Reo‐induced cytokine responses efficiently suppress HCV replication. Therefore, Reo and other select proinflammatory OVs may be used in the treatment of various cancers and attenuate both virus‐associated oncogenic drive and tumor burden [95].

Adoptive cell therapy (ACT) is based on a variety of engineered cells, including T‐cell receptor T cells (TCR‐Ts), chimeric antigen receptor T cells (CAR‐Ts), and CAR‐NK cells [96, 97]. These cells present high specificity to recognize antigens localized in cancer cells. During production, T cells are collected from patients and engineered to express CARs specific for recognizing tumor‐specific antigens and then are readministered to patients. CAR‐T‐cell therapy was first described in 1993. CAR‐T cells can recognize targets on cancer cells and then present antitumor effects through the release of inflammatory cytokines. Currently, ICB and CAR‐T‐cell therapy are viable therapeutic strategies for patients with various cancers, especially GC. The predominant targets of CAR‐T therapy are B‐cell surface antigens, such as the extracellular glycoproteins CD19 and CD20 [98, 99]. Therefore, CAR‐T therapy has yielded unprecedented efficacy in treating B‐cell malignancies [100, 101]. Moreover, a variety of cancer patients, including those with glioma, gastrointestinal cancer, and lung cancer, exhibit certain benefits from adoptive cell therapies [102, 103, 104]. In addition, unlike MHC‐independent CARs, TCRs respond to tumor‐associated intracellular antigens presented by MHCs [105]. Moreover, another type of ACT, which includes tumor‐infiltrating immune cell therapy, cytokine‐induced killing cell therapy, lymphocyte cytokine‐activated killing cell therapy, and NK‐cell therapy, is based on patient‐derived peripheral blood or tumors *in situ* and further *in vitro* amplification and activation and then transfusion for antitumor therapies [106, 107].

## The regulatory function of cancer immunotherapies on oxidative stress

Despite the different mechanisms of these therapeutic strategies, both ICB and CAR‐T therapy affect the oxidative stress status in the TME. Moreover, the alteration of tumor redox status contributes to immunotherapeutic efficacy [108]. It has been revealed that a variety of cells, including fibroblasts, can modulate ROS in the TME and further facilitate the drug resistance of GC [109]. In addition, according to an experimental study, ACT can significantly affect tumor metabolism and the subsequent accumulation of ROS in tumor cells [110]. The TNF derived from T lymphocytes showed synergistic effects with chemotherapy and enhanced redox production in cancer cells, while the therapeutic effects of ACT could be inhibited by scavenging ROS. It has been demonstrated that chemotherapy‐induced tumor cell death is partially based on the depletion of GSH, which can be achieved by T lymphocyte‐based immunotherapy. CD4+ effector T cells can impair various metabolic pathways and induce deficits in GSH synthesis. Therefore, the ability of T lymphocytes to induce oxidative destruction is responsible for the efficacy of ACT [111]. Additionally, the influence of therapeutic antibodies on oxidative stress has been revealed in previous studies. By using a cocktail of immunomodulators, the tumor mass can be decreased by inhibiting cell proliferation. Notably, elevated oxidative stress, apoptosis, and immune infiltration can also be observed in the TME, indicating the causative role of therapeutic antibodies in oxidative stress, which is responsible for cell cycle arrest and cell death [88].

Recently, accumulating evidence has revealed the underlying mechanisms and alterations in oxidative stress involved in the dynamic interaction among cancer cells, immune cells, and other cells within the TME. Among them, T lymphocytes play a role in inducing oxidative stress in tumor cells and respond to immunotherapy for GC; however, they are also susceptible to the inhibitory function of ROS mediated by immunosuppressive cells [112]. Therefore, therapeutic strategies aiming to amplify oxidative stress in cancer cells and attenuate the immune regulatory function of ROS on T lymphocytes within the TME are needed.

## The translational importance of ROS‐modulating agents for cancer immunotherapy

Although targeting immunity within the TME is a promising therapeutic strategy for cancers, there are still several challenges in clinical practice in terms of efficacy and safety. According to clinical trials, CAR‐T cells against CD19 are effective in the treatment of acute lymphoblastic leukemia; however, patients with other cancers may experience limited benefits [113]. Notably, various side effects can be caused by immunotherapy that involves autoimmunity. Thus, the establishment of novel immunotherapy with high safety and ensured efficacy is an urgent need in clinical practice [114]. Due to the multifaceted function of oxidative stress in the regulation of immune responses in GC, targeting ROS‐induced oxidative stress in the TME may potentiate the efficacy of cancer immunotherapy.

**Fig. 2:**
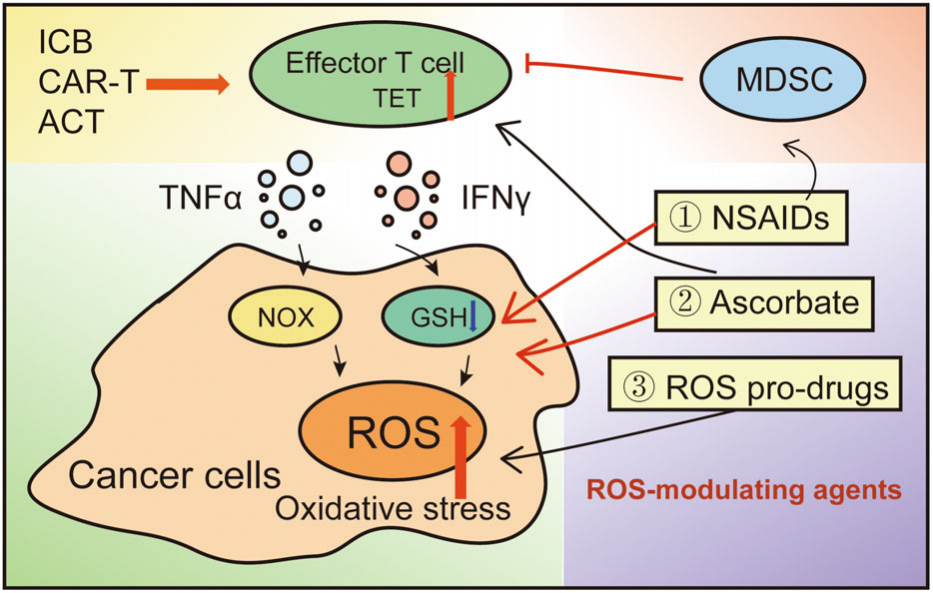
Underlying mechanisms of certain pro‐oxidants enhance the efficacy of cancer immunotherapies.

During the past decades, accumulating efforts have been made to establish novel approaches targeting the redox pathways in cancer cells. Although certain benefits can be observed with pro‐oxidant administration, the use of these compounds also encounters challenges in clinical practice due to their poor selectivity, their extensive toxicity, and tumor chemoresistance. Therefore, the combination of pro‐oxidants and immunotherapy may promote durable antitumor effects and minimize unwanted side effects. According to a previous study, elevated oxidative stress in cancer cells promotes the efficacy of ICB and ACT, suggesting that intensified tumor redox enhances sensitivity to immunotherapy [115]. In the following section, we emphasize some representative agents with potential promising clinical value to enhance the efficacy of T‐cell‐based immunotherapy. In this section, we introduce three predominant ROS‐modulating agents used for cancer immunotherapy, and the potential mechanisms are summarized in Fig. 2.

### Ascorbic acid and T‐cell‐based immunotherapies

Ascorbate, also known as vitamin C, has an antioxidative function at physiological doses. It has been reported that ascorbic acid serves as an antioxidant to protect GC cells against the toxic effects of ingested nitrosodimethylamines and heterocyclic amines. However, accumulating evidence has also demonstrated that a high dose of ascorbate can act as a pro‐oxidant to induce extracellular hydrogen peroxidation, leading to DNA damage [116]. Because the oral administration of ascorbate is associated with gut and renal filtration, the pharmacologic concentrations of ascorbate cannot be achieved; thus, intravenous administration is the most optimal approach. According to previous studies, high‐dose ascorbate has an antitumor function in the treatment of GC [116, 117]. Recently, there have been increasing numbers of clinical trials aiming to investigate the efficacy of high‐dose ascorbate in cancer treatment. Based on completed clinical trials, it has been proven that high‐dose ascorbate can attenuate the side effects caused by chemotherapy. Of note, high‐dose ascorbate can selectively destroy cancer cells with KRAS or BRAF mutations in human colorectal cancer (CRC). A previous study highlighted that GLUT1 is a marker for the sensitivity of GC to ascorbate and was associated with the antitumoral efficacy of therapeutic doses of ascorbate [118]. Increased uptake of the oxidized form of ascorbate leads to the accumulation of ROS in CRC cells with KRAS or BRAF mutations and their death and does not affect normal cells. Of note, ascorbate also exerts its antitumor function in a ROS‐independent manner. It has been demonstrated that ascorbate is a cofactor for the Ten‐eleven Translocation (TET) enzymes [119]. High‐dose ascorbate can reduce methylation in cancer cell lines via TET activation, which is independent of the induction of oxidative stress. Therefore, the pharmacological concentration of ascorbate exerts antitumor effects through both pro‐oxidative effects and epigenetic regulation. Regarding combination with immunotherapies, it has been revealed that the effects of high‐dose ascorbate synergize with those of ICB therapy in resident tumor‐bearing models. High‐dose ascorbate amplified the efficacy of ICB therapy treatment of various cancers, accounting for the enhanced infiltration of CD8+ T cells and IL‐12 production by APCs. Moreover, high‐dose ascorbate does not injure effector T cells but rather enhances immune functions through TET‐mediated epigenetic modifications [120].

### Nonsteroid anti‐inflammatory drugs and T‐cell‐based immunotherapies

Nonsteroid anti‐inflammatory drugs (NSAIDs) can inhibit the synthesis of prostaglandins and are commonly used for the relief of pain and inflammation in clinical practice [115]. Recently, several types of NSAIDs have been proven to suppress carcinogenesis and reduce the incidence of various cancers in humans, especially GC and CRC. Cyclooxygenases (COXs) promote the synthesis of prostaglandin E2 (PGE2), which can bind to receptors in cancer cells and further enhance cell proliferation, migration, and chemoresistance [121, 122]. Therefore, the antitumoral function of NSAIDs is partially based on the COX2/PGE2 axis; however, several additional mechanisms of NSAIDs have been investigated in recent studies. It has been reported that NSAIDs can trigger oxidative stress and then lead to the apoptosis of cancer cells. NSAID treatment destabilizes the redox balance and increases ROS production, which further results in the activation of the caspase cascade in cancer cells. To date, there are a variety of NSAIDs, including sulindac, celecoxib, and indomethacin, presenting antitumoral function mediated by the induced ROS in a COX‐independent manner in GC [123, 124, 125]. Notably, the antitumor process of NSAIDs also provokes the alteration of immune responses within the TME. PGE2 is a potent immunosuppressive factor enriched within the TME, while the inhibition of PGE2 by NSAIDs induces antitumor immunity.

Furthermore, NSAID‐induced oxidative stress is associated with ICD and tumor immunosurveillance [126]. After the administration of NSAIDs, tumor antigen‐specific T lymphocytes present stronger antitumor effects in GC [127]. According to another study, several types of NSAIDs augment the efficacy of anti‐PD‐1 therapy in the treatment of a variety of cancers, including GCs [128]. The above results prove the compatible function of NSAIDs with T‐cell‐based immunotherapies. Therefore, it is necessary to develop non‐COX inhibitory NSAIDs to prevent side effects, including gastrointestinal and cardiovascular toxicities, and meet the need for long‐term combination with immunotherapy.

### ROS‐responsive prodrugs and T‐cell‐based immunotherapies

Reactive oxygen species production is higher in cancer cells than in normal cells. Therefore, the establishment of prodrugs targeting only ROS‐enriched cells is another promising option to selectively promote ROS production in cancer cells and reduce toxicity to normal cells. ROS‐responsive prodrugs facilitate the delivery of therapeutic molecules into tumor cells, leading to the considerable inhibition of cell proliferation [129]. Recently, several studies have established novel prodrugs that serve as DNA cross‐linking or alkylating agents upon the production of ROS. Leinamycin (LNM), a potent antitumor antibiotic, can be activated by ROS and exhibit potent cytotoxicity in cancer cells with excessive ROS [130]. In addition, several aromatic nitrogen mustards can be released by the stimulation of boronates and H_2_O_2_ in cancer cells [131]. It has been reported that such prodrugs can selectively present cytotoxic functions against cancer cells with high levels of ROS in leukemia and breast cancer. Of note, such prodrugs are not harmful toward immune cells and normal cells at the doses required to destroy cancer cells, indicating their potential clinical value when used with immunotherapy. Moreover, ferrocene‐mediated oxidation has been applied to develop prodrugs with selective antitumor function [132]. These prodrugs present selective toxicity to cancer cells but maintain weak toxicity to normal cells. *N*‐(3‐(Piperidin‐1‐ylmethyl)benzyl)‐4‐(ferrocenylcarbamatmethyl)phenyl boronic acid pinacol ester (PipFcB), a newly developed prodrug, sensitized cancer cells to CD19 CAR‐T cells in human lymphoma cells or lymphocytic leukemia cells [133]. Additionally, the exposure of CAR‐T cells to PipFcB did not affect T‐cell exhaustion and viability, indicating the potential synergistic anticancer effects of combination with prodrugs with immunotherapies. Such mutual enhancement of the ROS‐induced loop results in specific antitumor function with minimized toxicities in normal cells, which may be a promising therapeutic strategy in clinical practice [133].

## Conclusion

Recently, accumulating evidence has demonstrated that immunotherapy‐induced ROS accumulation in cancer cells is not merely a metabolic byproduct but in turn contributes to the greater efficacy of immunotherapy. However, there have been limited clinical trials revealing the relationship between various immunotherapies and the production of oxidative stress in cancer cells. Several FDA‐approved drugs, such as ascorbate and NSAIDs, present a promising antitumor function when combined with immunotherapy treatment of various cancers. Moreover, with the advances in pro‐oxidants in recent decades, a multitude of novel T‐cell‐compatible agents have been established to enhance the efficacy of ICB or CAR‐T therapy, especially ROS‐responsive prodrugs. Therefore, the combination of ROS‐modulating agents and cancer immunotherapy may be a promising therapeutic strategy for treating various cancers in clinical practice.

Reactive oxygen species are critical signaling molecules that are continuously generated, transformed, and consumed in various biochemical reactions. Recently, accumulating evidence has revealed the critical role of ROS in the regulation of the immune response. Of note, the majority of immune cells are affected by the production of ROS and thus cannot normally exert their antitumor effect within the TME, leading to resistance to immunotherapy. Several ROS‐targeting strategies facilitate the development of multimodal immunotherapy strategies for cancer treatment. For instance, immunotherapy based on ROS‐producing drugs simultaneously enhances immunity against primary and metastatic tumors [134]. Therefore, immunotherapy combined with a ROS‐targeting strategy seems to be a promising antitumor strategy. In addition, it is well known that T lymphocytes are sensitive to oxidative stress; therefore, there are strict criteria for the pro‐oxidant option to assist immunotherapy; these methods should exhibit (a) limited effects on T lymphocytes and adaptive function in cancer cells; (b) easy administration and sufficient biosafety.

To further investigate the roles of oxidative stress‐related genes or pathways involved in the TME of GC, several available resources, such as databases or other tools, can be used for further exploration in this area. For example, oxidative stress‐related hub genes were identified by the STRING database (https://cn.string‐db.org/). The Human Protein Atlas (HPA) database (The Human Protein Atlas), which contains immunohistochemistry‐based expression data for GC, can be used to identify protein expression levels in tumor tissues. The correlation between OS time and hub gene expression, in addition to disease‐free survival time and hub gene expression, can be assessed by Kaplan–Meier survival analysis in the GEPIA database (http://gepia.cancer‐pku.cn/). Moreover, the correlations between the expression level of genes and immunoregulators, which contain immunoinhibitors, inmunostimulators, and MHC molecules, can be calculated by the TISIDB database (http://cis.hku.hk/TISIDB/). However, few studies have focused on ROS shuttling within the TME and the subsequent effects on immune cells. In addition, more clinical experimental data are required that focus on the safety and efficacy of combined therapeutic strategies. In this regard, the biological function and underlying mechanisms of ROS in different cells remain to be further explored. Taken together, these data suggest that more efforts should be made to determine the sequence and therapeutic window of the combination of ROS‐based agents and immunotherapy. The efficacy and biosafety also require further attention to establish novel approaches in the clinical treatment of GC.

## Conflict of interest

The authors declare no conflict of interest.

## Author contributions

YY and YW wrote the main manuscript text; YZ, ML, and XS prepared Figs 1 and 2. All authors reviewed the manuscript.

## Data Availability

The data will be available from the corresponding author upon reasonable request.
